# The homeobox gene *DLX4* regulates erythro-megakaryocytic differentiation by stimulating IL-1β and NF-κB signaling

**DOI:** 10.1242/jcs.168187

**Published:** 2015-08-15

**Authors:** Bon Q. Trinh, Nicolas Barengo, Sang Bae Kim, Ju-Seog Lee, Patrick A. Zweidler-McKay, Honami Naora

**Affiliations:** 1 Department of Molecular and Cellular Oncology, University of Texas MD Anderson Cancer Center, 1515 Holcombe Boulevard, Box 108, Houston, TX 77030, USA; 2 Department of Systems Biology, University of Texas MD Anderson Cancer Center, 1515 Holcombe Boulevard, Box 950, Houston, TX 77030, USA; 3 Division of Pediatrics, University of Texas MD Anderson Cancer Center, 1515 Holcombe Boulevard, Box 853, Houston, TX 77030, USA

**Keywords:** Homeobox gene, Megakaryocyte, Erythroid, Cytokine, NF-κB

## Abstract

Megakaryocyte and erythroid development are tightly controlled by a repertoire of cytokines, but it is not clear how cytokine-activated signaling pathways are controlled during development of these two lineages. Here, we identify that expression of DLX4, a transcription factor encoded by a homeobox gene, increases during megakaryopoiesis but decreases during erythropoiesis. Enforced expression of DLX4 in CD34^+^ stem and progenitor cells and in bipotent K562 cells induced lineage markers and morphologic features of megakaryocytes and repressed erythroid marker expression and hemoglobin levels. Converse results were obtained when DLX4 was knocked down. Gene Ontology and Gene Set Enrichment Analyses of genome-wide changes in gene expression revealed that DLX4 induces a megakaryocytic transcriptional program and inhibits an erythroid transcriptional program. DLX4 also induced gene signatures that are associated with nuclear factor κB (NF-κB) signaling. The ability of DLX4 to promote megakaryocyte development at the expense of erythroid generation was diminished by blocking NF-κB activity or by repressing *IL1B,* a transcriptional target of DLX4. Collectively, our findings indicate that DLX4 exerts opposing effects on the megakaryocytic and erythroid lineages in part by inducing IL-1β and NF-κB signaling.

## Abstract (precis)

**Summary:** The homeobox gene *DLX4* promotes megakaryocyte development at the expense of erythroid generation by inducing IL-1 and NF-κB signaling.

## INTRODUCTION

The megakaryocytic and erythroid lineages give rise to platelets and red blood cells, respectively. These two lineages are derived from the megakaryocyte-erythroid progenitor (MEP) cell and their development is dynamically controlled by a repertoire of cytokines. Thrombopoietin (TPO) and erythropoietin (EPO) have well-characterized functions in stimulating megakaryopoiesis and erythropoiesis, respectively (Kaushansky, 2006). Megakaryopoiesis is also augmented by interleukin (IL)-1 but is inhibited by platelet factor 4 (PF4) (Kimura et al., 1990; Lambert et al., 2007; van den Oudenrijn et al., 1999). By contrast, erythropoiesis is stimulated by activin A (ActA, a dimer composed of INHBA subunits) but is inhibited by tumor necrosis factor-α (TNF-α) (Roodman et al., 1987; Shiozaki et al., 1989; Yu et al., 1987). Specification of the megakaryocytic and erythroid lineages is orchestrated by a complex network of transcription factors. Several transcription factors such as GATA-1, FOG1 (also known as ZFPM1), SCL (also known as TAL1) and Gfi-1B are required for development of both the megakaryocytic and erythroid lineages (Hall et al., 2003; Randrianarison-Huetz et al., 2010; Takahashi et al., 1998; Tsang et al., 1997). Other transcription factors such as EKLF (also known as KLF1) and Ets-1 promote differentiation of one lineage at the expense of the other (Bouilloux et al., 2008; Lulli et al., 2006). More recently, bioinformatic and mathematical modeling studies have revealed that the transcriptional circuitry that controls hematopoietic cell fate decisions is tightly interconnected (Moignard et al., 2013; Novershtern et al., 2011). However, it is not clear how transcription factors that control megakaryocytic and erythroid lineage specification are interconnected with pathways that are activated by cytokine cues. We speculate that at least some transcription factors that control development of these lineages might modulate cytokine signaling loops.

The homeobox gene super-family comprises more than 200 genes that encode transcription factors with a conserved helix-turn-helix DNA-binding domain (McGinnis and Krumlauf, 1992). Homeobox genes are expressed in a temporally and spatially restricted manner, and control axial patterning and morphogenesis of virtually all organ systems (McGinnis and Krumlauf, 1992). Substantial evidence indicates that members of the HOX family of homeobox genes control expansion and/or differentiation of hematopoietic cell populations. For example, *HOXA10* stimulates expansion of myeloid progenitors, but blocks differentiation of the megakaryocytic, erythroid and B-cell lineages (Buske et al., 2001; Magnusson et al., 2007). *HOXA5* causes a shift toward myeloid differentiation and away from erythroid differentiation (Crooks et al., 1999). As compared to HOX genes, the functions of non-HOX homeobox genes in hematopoiesis are less characterized. One example is *MEIS1* which promotes commitment towards a MEP cell fate (Cai et al., 2012; Zeddies et al., 2014). Another example is *VENTX* which promotes myeloid differentiation at the expense of lymphopoiesis (Rawat et al., 2010). The mechanisms by which homeobox genes control distinct sets of hematopoietic cell populations are poorly understood as only a few bona fide transcriptional targets have been identified, and it is unclear how homeobox genes interact with other components of the circuitry that regulate these cell populations.

*DLX4* is a member of the DLX family of homeobox genes (Panganiban and Rubenstein, 2002). Other DLX family members have been found to control a wide range of developmental processes such as neurogenesis and limb patterning (Panganiban and Rubenstein, 2002), but the developmental function of *DLX4* is unclear. It has been reported that *DLX4* is expressed in the bone marrow (Haga et al., 2000), but the distribution of its expression among the hematopoietic cell lineages is not known. In this study, we identified that *DLX4* expression is elevated throughout megakaryopoiesis but is downregulated during erythropoiesis. We therefore hypothesized that DLX4 promotes megakaryocyte development at the expense of erythroid generation. Our studies demonstrate that DLX4 exerts opposing effects on the megakaryocytic and erythroid lineages, and that these effects of DLX4 are mediated in part through its induction of IL-1β and nuclear factor κB (NF-κB) signaling.

## RESULTS

### DLX4 expression is upregulated during megakaryopoiesis and downregulated during erythropoiesis

**Fig. 1.**
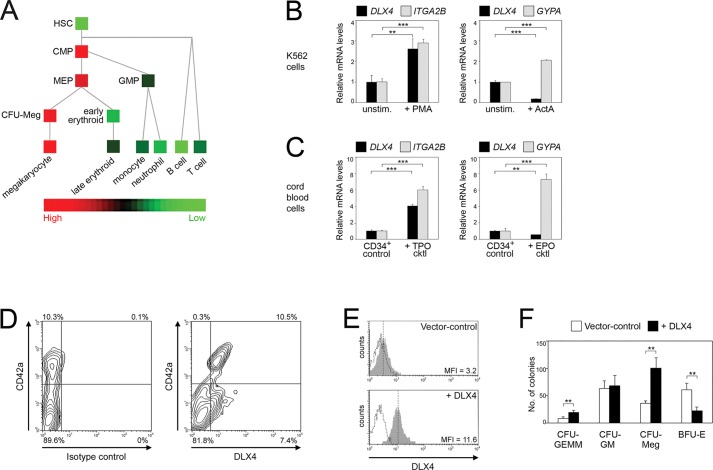
**Association of *DLX4* expression with increased megakaryopoiesis and decreased erythropoiesis.** (A) Heatmap representation of *DLX4* mRNA levels in stem, progenitor and mature human hematopoietic cell populations in the gene expression dataset of Novershtern et al. (Novershtern et al., 2011) (GEO Accession no. GSE24759). The labels of the indicated populations correspond to those used in the study of Novershtern et al. (2011) with the exception of the following: HSC (combination of HSC1 and HSC2), CFU-Meg (CFU-MK), early erythroid (ERY1), late erythroid (ERY5), B cell (naïve B cell), T cell (combination of naïve CD4^+^ and CD8^+^ T cells). (B) K562 cells were stimulated for 3 days with PMA (left panel) and with ActA (right panel) to induce megakaryocytic and erythroid differentiation, respectively. Shown are mRNA levels of *DLX4, ITGA2B* and *GYPA*, relative to the respective levels in unstimulated (unstim.) cells. (C) CD34^+^ cord blood cells were cultured for 5 days in medium supplemented with TPO cocktail (cktl) (left panel) and in medium supplemented with EPO cktl (right panel) to induce megakaryocytic and erythroid differentiation, respectively. Shown are mRNA levels of *DLX4, ITGA2B* and *GYPA*, relative to the respective levels in non-induced (control) cells. (D) CD34^+^ cells were cultured in medium supplemented with TPO cktl. After 5 days, cells were evaluated by flow cytometry for cell surface staining of CD42a and for intracellular staining of isotype control (left panel) or DLX4 (right panel). The percentages of cells in each quadrant are indicated. (E) CD34^+^ cells were transduced with GFP-expressing vector control and DLX4 (+DLX4) lentiviruses. Intracellular staining of DLX4 was evaluated by flow cytometry within the gated population of transduced GFP^+^ cells. Solid gray histograms represent staining with DLX4 antibody with mean fluorescence intensities (MFI) indicated. Dotted histograms represent staining with isotype control. (F) Transduced CD34^+^ cells were sorted for GFP and then seeded in semi-solid medium. After 2 weeks, colonies that originated from 10^4^ GFP-sorted cells were scored. Shown in B, C and F are mean±s.d. values of three independent experiments. **P<0.01, ***P<0.001.

We initially evaluated the distribution of *DLX4* expression in hematopoietic cell lineages by analyzing the gene expression data of cell populations that were directly isolated from human blood from the study of Novershtern et al. (Novershtern et al., 2011). *DLX4* mRNA levels were low in hematopoietic stem cells (HSCs) but were elevated in common myeloid progenitor (CMP) and MEP cells (Fig. 1A). *DLX4* mRNA levels remained elevated throughout megakaryocyte development but were markedly downregulated in the erythroid lineage (Fig. 1A).

To confirm the difference in *DLX4* expression in cells undergoing megakaryocytic and erythroid differentiation, we evaluated *DLX4* mRNA levels in K562 cells. K562 cells are widely used as a bipotent model and undergo megakaryocytic differentiation when stimulated by phorbol-12-myristate-13-acetate (PMA) and erythroid differentiation when stimulated by ActA (Whalen et al., 1997; Yu et al., 1987). *DLX4* expression in K562 cells significantly increased during PMA-induced megakaryocytic differentiation (*P*<0.01) (Fig. 1B, left panel), and decreased during ActA-induced erythroid differentiation (*P*<0.001) (Fig. 1B, right panel). mRNA levels of *ITGA2B* (encoding CD41) and *GYPA* (encoding glycophorin A or GYPA) were assayed as controls for megakaryocytic and erythroid differentiation, respectively (Fig. 1B). We confirmed our findings by using normal CD34^+^ cord blood stem and progenitor cells that were induced to undergo megakaryocytic and erythroid differentiation by stimulation with medium containing TPO and EPO, respectively. *DLX4* mRNA levels increased fourfold following induction of megakaryocytic differentiation (*P*<0.001) (Fig. 1C, left panel), but decreased 50% following induction of erythroid differentiation (*P*<0.01) (Fig. 1C, right panel). To confirm that the DLX4 protein level is increased in megakaryocytes, CD34^+^ cells were stimulated with medium containing TPO and thereafter evaluated for staining of DLX4 and the late megakaryocyte marker CD42a (also known as GP9). Almost all cells within the CD42a^+^ population (i.e. 97%) showed positive intracellular staining of DLX4 (Fig. 1D, right panel).

Because *DLX4* expression is upregulated in cells undergoing megakaryocytic differentiation but is downregulated in cells undergoing erythroid differentiation, we investigated the possibility that DLX4 promotes megakaryocyte development at the expense of erythroid generation. CD34^+^ cells were transduced with DLX4-expressing lentivirus (+DLX4) to produce a nearly fourfold increase in DLX4 levels (Fig. 1E). Equivalent numbers of vector control and +DLX4 CD34^+^ cells were seeded in semi-solid medium and assayed for colony formation. As compared to vector control cells, +DLX4 cells formed higher numbers of multipotent granulocyte, erythrocyte, macrophage, megakaryocyte colony-forming units (CFU-GEMM) and similar numbers of granulocyte-macrophage colony-forming units (CFU-GM) (Fig. 1F). Notably, +DLX4 cells formed significantly higher numbers of megakaryocyte colony-forming units (CFU-Meg) (*P*<0.01), but fewer numbers of erythroid-burst-forming units (BFU-E) (*P*<0.01) (Fig. 1F).

### DLX4 induces cellular features of megakaryocytes

**Fig. 2.**
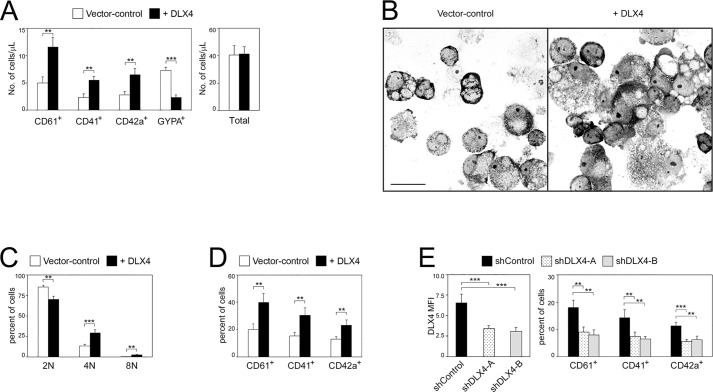
**DLX4 stimulates generation of cells with megakaryocytic features.** (A) Vector control and DLX4-transduced CD34^+^ cells were propagated in liquid cultures that contained both TPO and EPO cocktails and supported expansion of both the megakaryocytic and erythroid lineages. After 5 days, absolute numbers of transduced GFP^+^ cells that expressed lineage markers were evaluated by flow cytometry using counting beads. In B, C and D, vector control and DLX4-transduced CD34^+^ cells were sorted for GFP and cultured in medium containing TPO cocktail. At 5 days thereafter, cells were stained and analyzed as follows. (B) Morphology of representative cytospin preparations of cells stained with Wright Giemsa solution. Scale bar: 20 µm. (C) DNA content was evaluated by flow cytometric analysis of propidium iodide staining. Average percentages of cells with different ploidies are indicated. (D) Average percentages of cells with positive staining of megakaryocytic markers. (E) CD34^+^ cells were transduced with GFP-expressing lentiviruses that co-expressed non-targeting shRNA (shControl) or shRNAs that targeted two different sites of DLX4 (shDLX4-A, shDLX4-B) and were then cultured in medium containing TPO cocktail. After 5 days, transduced GFP^+^ cells were evaluated by flow cytometry for staining of DLX4 (left panel) and of megakaryocytic markers (right panel). Shown in A and C– E are mean+s.d. values of three independent experiments. **P<0.01, ***P<0.001.

We sought to confirm that DLX4 promotes megakaryocyte development at the expense of erythroid generation by evaluating the absolute numbers of megakaryocytic and erythroid cells. Vector control and +DLX4 CD34^+^ cord blood cells were propagated in liquid cultures that contained both TPO and EPO and that supported expansion of both lineages. Thereafter, absolute numbers of lineage-positive cells were quantified by flow cytometry using counting beads. Enforced expression of DLX4 significantly increased the numbers of cells that expressed the megakaryocytic markers CD61 (also known as ITGB3), CD41 and CD42a (*P*<0.01), but decreased the numbers of cells that expressed the erythroid marker GYPA (*P*<0.001) (Fig. 2A).

To evaluate the effect of DLX4 on cellular features of megakaryocytes derived from CD34^+^ cells, transduced CD34^+^ cells were stimulated with medium containing TPO. Following stimulation, +DLX4 cells exhibited more prominent multi-lobulated nuclei and increased cell size than vector control cells (Fig. 2B). Cord-blood-derived megakaryocytes exhibit little polyploidy, with ∼80% of these cells having a 2N content of DNA (Mattia et al., 2002). We observed a similar DNA content in vector control cells (Fig. 2C). By contrast, polyploidy increased when DLX4 was expressed (*P*<0.01) (Fig. 2C). Enforced expression of DLX4 also increased the proportion of cells that expressed megakaryocytic markers (*P*<0.01) (Fig. 2D). In converse experiments, we evaluated whether repressing DLX4 prevents CD34^+^ cells from undergoing megakaryocytic differentiation by transducing CD34^+^ cells with *DLX4*-targeting short hairpin RNAs (shRNAs) (Fig. 2E, left panel). Knockdown of DLX4 significantly decreased the proportion of cells that expressed megakaryocytic markers (*P*<0.01) (Fig. 2E, right panel).

**Fig. 3.**
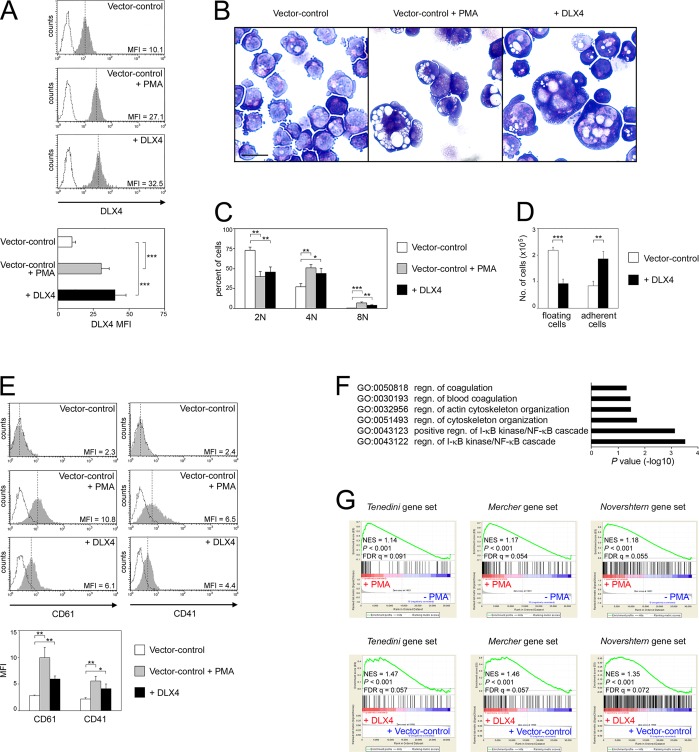
**DLX4 induces a megakaryocyte-specific transcriptional program.** Cellular features and gene expression profiles were analyzed in stable vector control and DLX4-overexpressing (+DLX4) K562 cell lines. Vector control K562 cells that were stimulated for 3 days with PMA (vector control+PMA) were used as a positive control for megakaryocytic differentiation. (A) Upper panel, representative examples of flow cytometric analysis of DLX4 staining (solid gray histograms with MFI indicated). Dotted histograms represent staining with isotype control. Lower panel, Average MFI of DLX4 staining. (B) Morphologic features of cells stained with Wright Giemsa solution. Scale bar: 20 µm. (C) Analysis of DNA content. (D) Equivalent numbers of cells (4×10^5^) were seeded in fibronectin-coated dishes. After 16 h, numbers of floating and adherent cells were counted. (E) Upper panel, representative examples of flow cytometric analysis of CD61 and CD41 staining (solid gray histograms with MFI indicated). Dotted histograms represent staining with isotype control. Lower panel, Average MFI of staining. (F) Comparison of global gene expression profiles of vector control and +DLX4 K562 cell lines revealed a total of 426 genes that are significantly upregulated in the +DLX4 line as compared to the vector control line. These upregulated genes were evaluated by Gene Ontology analysis. Gene groups associated with regulation (regn) of biological processes are indicated. (G) Lower panel, global changes in gene expression that are induced by DLX4 in K562 cells were evaluated by GSEA for enrichment of megakaryocyte gene sets from three independent studies (Mercher et al*.,* 2008; Novershtern et al*.,* 2011; Tenedini et al*.,* 2004). Normalized enrichment scores (NES), significance values and false discovery rates (FDR) are indicated. Upper panel, as a positive control, global changes in gene expression that are induced in vector control K562 cells by PMA stimulation were evaluated for enrichment of megakaryocyte gene sets. Shown in A, C, D and E are mean+s.d. values of three independent experiments. *P<0.05, **P<0.01, ***P<0.001.

To further evaluate the effect of DLX4 on cellular features, DLX4 was stably expressed in K562 cells (+DLX4) to produce a threefold increase in DLX4 levels. This induction was similar to the increase in endogenous DLX4 levels observed in vector control K562 cells undergoing PMA-induced megakaryocytic differentiation (Fig. 3A). Enforced expression of DLX4 alone induced a dramatic increase in cell size and prominent multi-lobulated nuclei (Fig. 3B). The morphologic features induced by DLX4 were similar to those of vector control K562 cells undergoing PMA-induced megakaryocytic differentiation (Fig. 3B). Polyploidy in +DLX4 cells was significantly higher than that in unstimulated vector control cells (*P*<0.01) and was similar to that of PMA-stimulated vector control cells (Fig. 3C). Megakaryocytic differentiation is associated with increased cell adhesion (Machlus and Italiano, 2013). *In vitro* attachment assays revealed that +DLX4 K562 cells were more adhesive than vector control K562 cells (*P*<0.01) (Fig. 3D). Enforced expression of DLX4 alone also induced expression of megakaryocytic markers (Fig. 3E). In converse experiments, we evaluated the effects of knocking down DLX4 in K562 cells. Following PMA stimulation, levels of megakaryocytic markers and polyploidy were induced in control K562 cells but this induction was blocked in *DLX4* shRNA-transfected K562 cells (supplementary material Fig. S1A–D).

### DLX4 induces a megakaryocyte-specific transcriptional program

To investigate the ability of DLX4 to promote megakaryocytic differentiation at the molecular level, we analyzed genome-wide changes in gene expression that are induced by DLX4. The global gene expression profile of +DLX4 K562 cells was compared with that of unstimulated vector control K562 cells. A random-variance *t*-test with a stringent significance threshold (*P*<0.001) and a global test with 1000 permutations were applied. A total of 707 differentially expressed genes were obtained (i.e. 426 upregulated genes and 281 downregulated genes in the +DLX4 line as compared to the vector control line). Gene Ontology analysis (Dennis et al., 2003) of genes that were upregulated in the +DLX4 line as compared to the vector control line revealed that DLX4 alone induces expression of groups of genes that are associated with megakaryocyte and platelet function (Fig. 3F). Consistent with the changes in cell size and structure induced by DLX4, DLX4 also induced expression of gene groups that are associated with cytoskeletal organization (Fig. 3F).

Global changes in gene expression that are induced by DLX4 were also analyzed by Gene Set Enrichment Analysis (GSEA) (Subramanian et al., 2005). Consistent with the increased cell adhesiveness of +DLX4 cells, DLX4 induced changes in gene expression that are enriched for gene sets associated with cell adhesion (supplementary material Table S1). Furthermore, DLX4 alone induced changes in gene expression that are enriched for two independent megakaryocyte gene sets (Fig. 3G, lower panel). The megakaryocyte gene set, described by Tenedini et al., includes genes that are essential for megakaryocyte development (Tenedini et al., 2004). The megakaryocyte gene set, described by Mercher et al., includes previously reported megakaryocyte-specific genes (Mercher et al., 2008). DLX4 also induced changes in gene expression that are enriched for a third gene set that we generated and that comprised genes that are upregulated in mature megakaryocytes in the dataset of Novershtern et al. (Novershtern et al., 2011) (Fig. 3G, lower panel). Levels of expression in vector control and +DLX4 K562 cells of ‘leading edge’ genes in the megakaryocytic gene set that we generated from the Novershtern et al. data are shown in supplementary material Fig. S2. As expected, global changes in gene expression that were induced in vector control K562 cells by PMA stimulation were enriched for the three megakaryocytic gene sets and were used as a positive control (Fig. 3G, upper panel). These findings indicate that DLX4 induces a megakaryocyte-specific transcriptional program.

### DLX4 represses an erythroid-specific transcriptional program

**Fig. 4.**
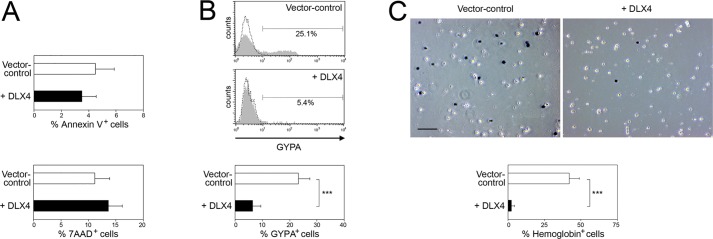
**DLX4 inhibits generation of cells with erythroid features.** (A) Vector control and DLX4-transduced CD34^+^ cells were cultured in medium containing EPO cocktail. After 5 days, transduced GFP^+^ cells were evaluated by flow cytometry for Annexin V and 7AAD staining. (B) Transduced cells were cultured as in A. Upper panel, representative examples of flow cytometric analysis of GYPA staining (solid gray histograms). Dotted histograms represent staining with isotype control. Lower panel, average percentages of GYPA^+^ cells. (C) Transduced CD34^+^ cells were sorted for GFP and then cultured as in A. Hemoglobin synthesis was assayed by benzidine staining. Stained (blue) and unstained (white) cells were counted in three random 100× microscopic fields per experiment. Upper panel, examples of cell staining. Scale bar: 100 µm. Lower panel, average percentages of stained cells per field. Shown in A, B and C are mean+s.d. values of three independent experiments. ***P<0.001.

We earlier observed that DLX4 increased the number of megakaryocytic cells and concomitantly decreased the number of erythroid cells when transduced CD34^+^ cells were propagated in medium that supported expansion of both lineages (Fig. 2A). Because it is possible that one cell population might shrink due to competitive expansion of another cell population, we tested whether DLX4 inhibits erythroid differentiation independently of its stimulatory effect on the megakaryocyte lineage. Transduced CD34^+^ cells were induced to undergo erythroid differentiation by stimulation with medium that contained EPO but no TPO. Enforced expression of DLX4 did not increase cell death as assayed by Annexin V and 7-aminoactinomycin D (7AAD) staining (Fig. 4A). However, DLX4 significantly decreased the percentages of cells that expressed GYPA (*P*<0.001) and hemoglobin (*P*<0.001) (Fig. 4B,C). These findings indicate that DLX4 blocks erythroid differentiation.

**Fig. 5.**
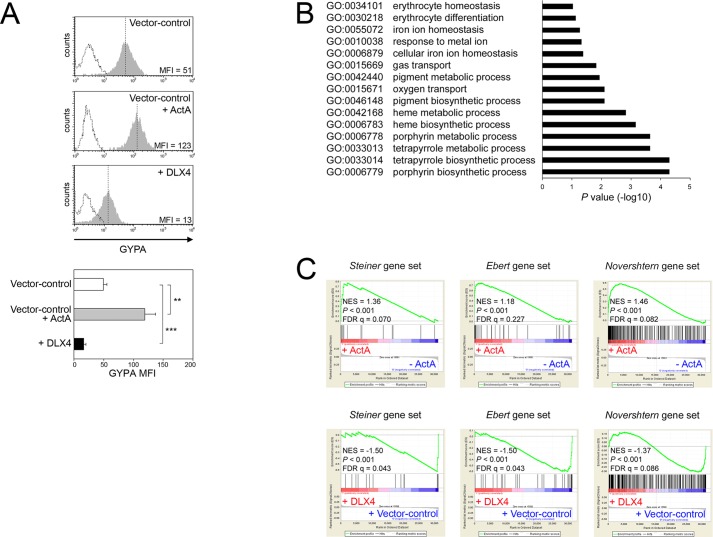
**DLX4 represses an erythroid transcriptional program.** (A) Upper panel, representative examples of flow cytometric analysis of GYPA staining in vector control and +DLX4 K562 cells (solid gray histograms with MFI indicated). Dotted histograms represent staining with isotype control. Vector control K562 cells that were stimulated for 3 days with ActA (vector control+ActA) were used as a positive control for erythroid differentiation. Lower panel, average MFI of GYPA staining of three independent experiments. **P<0.01, ***P<0.001. (B) Comparison of global gene expression profiles of vector control and +DLX4 K562 cell lines revealed a total of 281 genes that are significantly downregulated in the +DLX4 line as compared to the vector control line. These downregulated genes were evaluated by Gene Ontology analysis. Gene groups associated with regulation of biological processes are indicated. (C) Lower panel, global changes in gene expression that are induced by DLX4 in K562 cells were evaluated by GSEA for enrichment of erythroid gene sets from three independent studies (Ebert et al*.,* 2008; Novershtern et al*.,* 2011; Steiner et al*.,* 2009). Upper panel, as a positive control, global changes in gene expression that are induced in vector control K562 cells by ActA stimulation were evaluated for enrichment of erythroid gene sets.

We further investigated the ability of DLX4 to inhibit erythroid differentiation by evaluating gene expression changes in K562 cells. Vector control K562 cells that were stimulated with ActA were used as a positive control for erythroid differentiation. GYPA levels in K562 cells significantly decreased when DLX4 was overexpressed (*P*<0.001) (Fig. 5A). Conversely, GYPA levels increased when endogenous DLX4 was knocked down (*P*<0.01) (supplementary material Fig. S3). Gene Ontology analysis of genes that are downregulated in the +DLX4 K562 line, as compared to the vector control K562 line, revealed that DLX4 represses groups of genes that are associated with erythroid development and function such as heme synthesis and oxygen transport (Fig. 5B). Furthermore, GSEA revealed that DLX4 induced global changes in gene expression that were inversely correlated with two independent erythroid gene sets (Fig. 5C, lower panel). The erythroid gene set, described by Steiner et al., includes erythrocyte membrane protein genes (Steiner et al., 2009). The erythroid gene set, described by Ebert et al., includes genes that are coordinately expressed during *in vitro* erythroid differentiation of human CD34^+^ adult bone marrow cells (Ebert et al., 2008). Inverse correlation was also found with a third gene set that we generated and that comprised genes that were upregulated in late erythroid cells in the dataset of Novershtern et al. (2011) (Fig. 5C, lower panel). Levels of expression in vector control and +DLX4 K562 cells of ‘leading edge’ genes in the erythroid gene set that we generated from the Novershtern et al. data are shown in supplementary material Fig. S2. In contrast, changes in gene expression that were induced in vector control K562 cells by ActA stimulation were found, as expected, to be enriched for the three erythroid gene sets (Fig. 5C, upper panel). These findings indicate that DLX4 represses an erythroid-specific transcriptional program.

### DLX4 stimulates NF-κB signaling

**Fig. 6.**
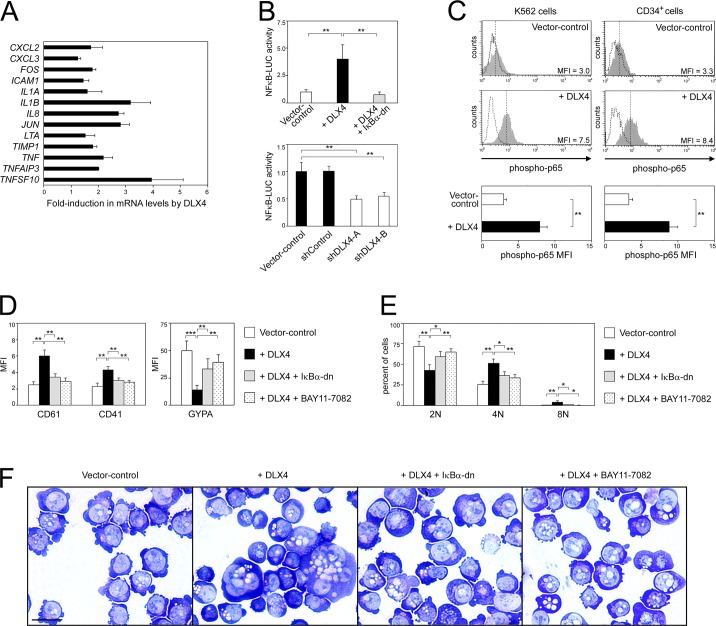
**DLX4 stimulates NF-κB signaling.** (A) Expression of NF-κB target genes was assayed in vector control and +DLX4 K562 cells by qRT-PCR. Shown is the average fold induction in the mRNA level of each gene in +DLX4 cells relative to its respective level in vector control cells. (B) NF-κB-LUC reporter activity was assayed in vector control K562 cells and in +DLX4 K562 cells that lacked or expressed ΙκΒα-dn (upper panel), and also in K562 cells that were transfected with non-targeting shRNA (shControl) and with two different *DLX4* shRNAs (shDLX4-A, shDLX4-B) (lower panel). (C) Upper panel, representative examples of flow cytometric analysis of intracellular staining of phosphorylated NF-κB p65 in K562 cells and in CD34^+^ cells (solid gray histograms with MFI indicated). Dotted histograms represent staining with isotype control. Lower panel, average MFI of staining. In D, E and F, lineage markers, DNA content and morphology were evaluated in vector control K562 cells, in +DLX4 K562 cells that lacked or expressed ΙκΒα-dn, and in +DLX4 K562 cells that were treated with the IκB kinase inhibitor BAY11-7082 (1 µM) for 3 days. (D) Average MFI of CD61, CD41 and GYPA staining detected by flow cytometry. (E) Analysis of DNA content. (F) Morphologic features of K562 cells stained with Wright Giemsa solution. Scale bar: 20 µm. Shown in A–E are mean±s.d. values of three independent experiments. *P<0.05; **P<0.01, ***P<0.001.

Gene Ontology analysis of genes that are upregulated in the +DLX4 K562 line as compared to the vector control K562 line revealed that DLX4 induces expression of groups of genes that are associated with the canonical NF-κB signaling pathway (Fig. 3F). GSEA also revealed that DLX4 induces changes in gene expression that are enriched for NF-κB-associated gene sets in the Broad Institute Molecular Signature Database (supplementary material Table S2). Quantitative real-time PCR (qRT-PCR) analysis confirmed that DLX4 induces expression of several known NF-κB target genes in K562 cells (Fig. 6A) and in CD34^+^ cells (supplementary material Fig. S4A). To evaluate the effect of DLX4 on NF-κB activity, we assayed the activity of a luciferase reporter construct driven by tandem NF-κB-binding sites (NF-κB-LUC). NF-κB-LUC activity in K562 cells was induced when DLX4 was overexpressed (*P*<0.01) (Fig. 6B, upper panel) and, conversely, was inhibited when endogenous DLX4 was knocked down (*P*<0.01) (Fig. 6B, lower panel). In the canonical NF-κB pathway, transcriptional activity of NF-κB is stimulated upon phosphorylation of its p65 (also known as RelA) subunit (Perkins, 2012). DLX4 increased phosphorylation of p65 in K562 cells and in CD34^+^ cells (Fig. 6C). These findings indicate that DLX4 stimulates NF-κB activity.

We subsequently investigated whether the effects of DLX4 on megakaryocytic and erythroid differentiation depend on NF-κB. We evaluated the effect of blocking canonical NF-κB signaling in +DLX4 K562 cells by expressing a dominant-negative mutant form of ΙκΒα (ΙκΒα-dn). ΙκΒα-dn contains S32A and S36A mutations that render it resistant to degradation and able to sequester NF-κB in the cytoplasm (Boehm et al., 2007). ΙκΒα-dn abrogated NF-κB-LUC activity in +DLX4 K562 cells (Fig. 6B, upper panel). Expression of ΙκΒα-dn in +DLX4 K562 cells relieved the repression of GYPA expression (*P*<0.01) (Fig. 6D, right panel) and also prevented the induction of CD61 and CD41 (*P*<0.01) (Fig. 6D, left panel). ΙκΒα-dn also diminished polyploidy and megakaryocytic morphology of +DLX4 K562 cells (Fig. 6E,F). To confirm our findings, we treated +DLX4 K562 cells with BAY11-7082, an agent that blocks NF-κB signaling by inhibiting the IκB kinase complex (Pierce et al., 1997). Treatment with BAY11-7082 inhibited the stimulatory effect of DLX4 on megakaryocytic differentiation and relieved the repressive effect of DLX4 on erythroid differentiation (Fig. 6D–F). Similar findings were observed when DLX4-transduced CD34^+^ cells were treated with BAY11-7082 (supplementary material Fig. S4B). Taken together, these findings indicate that DLX4 mediates its opposing effects on megakaryocytic and erythroid differentiation, at least in part, in an NF-κB-dependent manner.

### DLX4 mediates its effects on cell differentiation through induction of its transcriptional target *IL1B*

**Fig. 7.**
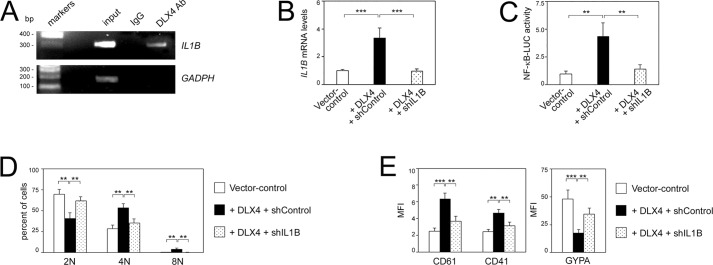
**DLX4 mediates its effects on megakaryocytic and erythroid differentiation by inducing its transcriptional target *IL1B*.** (A) Detection of binding of endogenous DLX4 in parental K562 cells to the *IL1B* promoter by chromatin immunoprecipitation. *GAPDH* was amplified as an irrelevant gene control. (B) qRT-PCR analysis of relative *IL1B* mRNA levels in vector control K562 cells and in +DLX4 K562 cells that were transfected with shControl and *IL1B* shRNAs. (C) NF-κB-LUC reporter activity in vector control K562 cells and in shRNA-transfected +DLX4 K562 cells. (D) Analysis of DNA content. (E) Average MFI of CD61, CD41 and GYPA staining detected by flow cytometry. Shown in B–E are mean±s.d. values of three independent experiments. **P<0.01, ***P<0.001.

DLX4 interacts with, and modulates the activity of, several transcription factors such as Smad4 and Sp1 (Trinh et al., 2011). However, interactions of DLX4 with the p65, c-Rel, p50 and p52 NF-κB subunits were not detected in immunoprecipitation assays using K562 cell lysates (data not shown), indicating that DLX4 stimulates NF-κB activity by other mechanisms. IL-1β is a potent stimulator of NF-κB activity (Perkins, 2012). Because DLX4 induces *IL1B* expression (Fig. 6A), we searched for DLX4-binding motifs in the *IL1B* promoter and identified a putative binding site at positions −359 to −353. Binding of endogenous DLX4 to this region was detected by chromatin immunoprecipitation (Fig. 7A). To determine whether DLX4 stimulates NF-κB activity by inducing *IL1B*, we knocked down the *IL1B* expression level in +DLX4 K562 cells to almost the level seen in vector control K562 cells (Fig. 7B). This knockdown reduced NF-κB-driven promoter activity in +DLX4 cells to a level comparable to that in vector control cells (Fig. 7C), indicating that DLX4 primarily stimulates NF-κB activity by inducing *IL1B* expression.

To determine whether DLX4 mediates its opposing effects on erythroid and megakaryocytic differentiation by inducing *IL1B*, we evaluated the effect of knocking down IL-1β in +DLX4 K562 cells. Knockdown of IL-1β reduced polyploidy in +DLX4 K562 cells (*P*<0.01) (Fig. 7D). Knockdown of IL-1β in +DLX4 K562 cells also relieved the repression of GYPA expression (*P*<0.01) (Fig. 7E, right panel), and prevented the induction of CD61 and CD41 (*P*<0.01) (Fig. 7E, left panel). To confirm our findings, we evaluated the effects of adding IL-1β neutralizing monoclonal antibody (mAb) to DLX4-transduced CD34^+^ cells that were propagated in medium that supported generation of both lineages. Treatment with IL-1β mAb significantly decreased the proportion of cells that expressed megakaryocytic markers (*P*<0.01) and restored the proportion of GYPA^+^ cells (*P*<0.001) (supplementary material Fig. S4C). Collectively, our findings indicate that DLX4 exerts opposing effects on the megakaryocytic and erythroid lineages, at least in part, in an NF-κB-dependent manner, and that this is mediated by its activation of its transcriptional target *IL1B*.

## DISCUSSION

Although it is widely recognized that development of hematopoietic cell lineages is controlled by cytokines and transcription factors, the relative roles of these regulators have been extensively debated. On the one hand, cell identity is thought to be dictated by stochastic variation in the balance of lineage-specifying transcription factors (Sarrazin and Sieweke, 2011). On the other hand, there is evidence that cytokines instruct lineage choice (Mossadegh-Keller et al., 2013; Rieger et al., 2009). Increasingly, the stochastic and instructive models are thought to be integrated and not mutually exclusive (Palani and Sarkar, 2009; Sarrazin and Sieweke, 2011). Several studies indicate that cytokines instruct cell fate changes by activating lineage-specifying transcription factors. For example, granulocyte colony-stimulating factor (G-CSF) signaling stimulates expression of C/EBPα which is crucial for neutrophil development (Dahl et al., 2003). Macrophage colony-stimulating factor (M-CSF) signaling induces expression of PU.1 (also known as SPI1) which is required for myeloid lineage commitment (Mossadegh-Keller et al., 2013). Reciprocally, transcription factors can direct sensitivity to cytokine signals by controlling expression of cytokine receptors. PU.1 activates transcription of genes encoding the receptors for M-CSF, G-CSF and granulocyte-macrophage colony-stimulating factor-α (GM-CSFα) (Hohaus et al., 1995; Smith et al., 1996; Zhang et al., 1994). GATA-1 activates transcription of the gene encoding the EPO receptor (Zon et al., 1991). In this study, we identified that the transcription factor DLX4 promotes megakaryocyte development at the expense of erythroid generation, and that these opposing effects of DLX4 are mediated in part through its transcriptional activation of *IL1B* which in turn stimulates the NF-κB signaling pathway. These findings support a model in which cell lineage choice is controlled by cytokines and lineage-specifying transcription factors that are interconnected through transcriptional control of cytokine signaling loops.

Our findings that DLX4 exerts opposing effects on the megakaryocytic and erythroid lineages at least in part by transcriptional activation of *IL1B* is supported by studies demonstrating that IL-1 augments megakaryopoiesis and represses erythropoiesis (Kimura et al., 1990; van den Oudenrijn et al., 1999; Means et al., 1992). In particular, our finding that DLX4 increases the number of megakaryocyte colony-forming units is consistent with a report that IL-1 is required along with TPO for optimally stimulating expansion of megakaryocyte progenitors (van den Oudenrijn et al., 1999). In addition, our findings that DLX4 skews development in favor of the megakaryocyte lineage at the expense of erythroid generation in an NF-κB-dependent manner is consistent with the ability of IL-1β to activate NF-κB and the reported ability of NF-κB to repress expression of erythroid-specific genes (Liu et al., 2003). Our finding that DLX4 inhibits the number of erythroid-burst-forming units is also supported by a report that erythroid burst formation is inhibited by TNF-α, a transcriptional target of NF-κB (Roodman et al., 1987). Our findings do not rule out the possibility that DLX4 might additionally control differentiation through other pathways that are activated by IL-1β, such as those involving mitogen-activated protein kinases. ERK signaling stimulates megakaryocytic differentiation, whereas loss of ERK1 causes expansion of erythroid progenitors (Guihard et al., 2010; Whalen et al., 1997). However, p38 signaling inhibits megakaryocytic differentiation (Chang et al., 2010). The stimulatory effects of IL-1β-mediated NF-κB and ERK signaling on megakaryocytic differentiation might therefore outweigh the inhibitory effects of IL-1β-mediated p38 signaling.

Because we observed that inhibition of either IL-1β or NF-κB substantially, but not completely, blocked the effects of DLX4 on megakaryocytic and erythroid differentiation, it is likely that DLX4 largely mediates its effects on these lineages by stimulating IL-1 and NF-κB signaling but can also partially mediate its effects in a IL-1- and NF-κB-independent manner. In regard to the latter, one mechanism might involve blocking ActA signal transduction. ActA stimulates expansion of erythroid progenitors (Shiozaki et al., 1989). We previously identified that DLX4 binds and inhibits Smad4 which is utilized by ActA for signal transduction (Trinh et al., 2011). BP1 has been reported to be an isoform of DLX4 and to repress the β-globin promoter (Chase et al., 2002). Different isoforms of DLX4 might therefore inhibit erythroid development by distinct mechanisms. The effect of BP1 on the megakaryocytic lineage is not known. Chen et al. (2014) recently identified that a splice variant isoform of the transcription factor NFIB promotes megakaryocyte differentiation, whereas the canonical NFIB isoform has no effect. Similarly, different isoforms of DLX4 might exert differing effects on the megakaryocytic lineage. Taken together with these previous reports, our study suggests a model in which transcription factors can exert stimulatory or antagonistic effects on different lineages by modulating distinct sets of signaling pathways and that this control might be tuned by the utilization of different transcription factor isoforms.

The mechanisms by which homeobox genes control hematopoiesis are poorly understood because only few transcriptional targets have been identified. One notable example is HOXA9 which promotes expansion of HSCs and myeloid progenitor cells, and activates transcription of the gene encoding fms-related tyrosine kinase-3 (FLT3) (Gwin et al., 2010). HOXA10 blocks erythroid and megakaryocytic differentiation and has been found to repress *GATA1* expression (Magnusson et al., 2007). Two studies identified that MEIS1 promotes commitment towards a MEP cell fate but have conflicting findings regarding the effect of MEIS1 on erythroid progenitors (Cai et al., 2012; Zeddies et al., 2014). Cai et al. (2012) also identified that MEIS1 increases megakaryocyte colony-forming unit generation. This finding is intriguing because MEIS1 activates the promoter of the gene encoding PF4 (Okada et al., 2003). PF4 is a chemokine that is expressed in megakaryocytes and platelets but negatively regulates megakaryopoiesis (Lambert et al., 2007). The mechanisms that regulate homeobox gene expression in hematopoietic cells are unclear. There is evidence that lineage-specifying transcription factors and cytokine signals form feedback loops. M-CSF signaling induces expression of PU.1 that in turn activates transcription of gene encoding the M-CSF receptor (Mossadegh-Keller et al., 2013; Zhang et al., 1994). G-CSF signaling induces expression of C/EBPα which controls G-CSF receptor expression (Dahl et al., 2003; Smith et al., 1996). We previously identified that DLX4 blocks transforming growth factor-β (TGF-β)–Smad-mediated gene expression and that TGF-β downregulates DLX4 expression in epithelial cells (Trinh et al., 2011). In this study, we similarly observed that DLX4 expression is downregulated by ActA. Given our finding that DLX4 stimulates IL-1 and NF-κB signaling, it is possible that DLX4 is reciprocally induced by IL-1 and NF-κB signaling in the megakaryocytic lineage.

In summary, the present study demonstrates that the homeobox gene *DLX4* promotes megakaryocytic development at the expense of erythroid generation, and that the opposing effects of DLX4 on these lineages are mediated in part through its ability to stimulate IL-1 and NF-κB signaling. To our knowledge, our study is the first to identify a regulatory relationship between a homeobox gene and NF-κB signaling in controlling hematopoietic cell differentiation. Our findings support increasing evidence that lineage-specifying transcription factors and cytokine signals are interconnected. Further investigation of the crosstalk between the pathways that control megakaryocytic and erythroid development will provide important insights for understanding the pathogenesis of diseases that involve defects in these lineages and for improving transfusion medicine.

## MATERIALS & METHODS

### Reagents

Sources of antibodies were as follows: DLX4 (Abcam, Cambridge, MA); phosphorylated p65 (Ser536) (Cell Signaling Technology, Danvers, MA); GYPA, CD41 (AbD Serotec, Kidlington, U.K.); CD42a (Miltenyi Biotec, San Diego, CA); IL-1β, CD61, fluorochrome-conjugated secondary antibodies (BD Biosciences, San Jose, CA). Sources of other reagents were as follows: recombinant human EPO, ActA, holo-transferrin, PMA, BAY11-7082 (Sigma-Aldrich, St Louis, MO); recombinant human stem cell factor (SCF), FLT3 ligand (FLT3L), TPO, IL-3 and IL-6 (Peprotech, Rocky Hill, NJ).

### Plasmids

A cDNA that encodes FLAG-tagged DLX4 (based on transcript variant 1, Genbank Accession no. NM_138281) was subcloned into the pIRES-EGFP2 (Clontech, Mountain View, CA) and pCDH-MSCV-MCS-EF1-copGFP (System Biosciences, Mountain View, CA) vectors. pMD2.G (VSV-G envelope) and psPAX2 (Gag-Pro-Pol) plasmids were provided by Didier Trono (Ecole Polytechnique Federale de Lausanne, Lausanne, Switzerland; Addgene plasmids 11259, 12260). pGIPZ plasmids containing non-targeting, *DLX4* and *IL1B* shRNAs were purchased from the MD Anderson Cancer Center shRNA and ORFeome Core Facility (Houston, TX), respectively. The ΙκΒα-dn construct (Boehm et al., 2007) was provided by William Hahn (Dana Farber Cancer Institute, Boston, MA; Addgene plasmid 15291). Viral vectors used in this study co-expressed GFP.

### Culture and transfection of cell lines

K562 and 293T packaging cells were purchased from American Type Culture Collection (Manassas, VA) and were cultured in RPMI 1640 medium and Dulbecco's modified Eagle's medium (DMEM; Cellgro, Manassas, VA), respectively. Media were supplemented with penicillin-streptomycin and 10% fetal bovine serum (FBS; Cellgro). K562 and 293T cells were transfected by using Lipofectamine 2000^®^ reagent (Invitrogen, Waltham, MA). Stably transfected cells were selected by G418 (400 μg/ml) (Invitrogen).

### Isolation and transduction of CD34^+^ cells

Cord blood units were obtained from the MD Anderson Cancer Center Cord Blood Bank under a protocol approved by the Institutional Review Board. Mononuclear cells were isolated from multiple units by density separation using Ficoll-Paque PREMIUM^®^ (GE Healthcare, Pittsburgh, PA) and pooled together. CD34^+^ cells were isolated from mononuclear cells by using CD34 microbeads (Miltenyi Biotec, San Diego, CA). For generating lentiviruses, 293T cells were co-transfected with viral expression and packaging plasmids. At 48 h thereafter, culture supernatants were harvested. Viruses were concentrated using PEG-it^®^ Virus Precipitation Solution (System Biosciences). CD34^+^ cells were pre-stimulated overnight in QBSF-60^®^ serum-free medium (Quality Biological, Gaithersburg, MD) supplemented with 100 ng/ml SCF, 100 ng/ml FLT3L and 100 ng/ml TPO, and then infected with lentiviruses using TransDux^®^ reagent (System Biosciences). At 24 h thereafter, cells were infected a second time and then incubated in QBSF-60^®^ serum-free medium supplemented with 50 ng/ml SCF, 80 ng/ml FLT3L, 50 ng/ml TPO and 100 ng/ml IL-6.

### Induction of differentiation in liquid cultures

To induce megakaryocytic differentiation of CD34^+^ cells, cells were cultured in StemSpan^®^ serum-free expansion medium (SFEM) (Stem Cell Technologies, Vancouver, Canada) that was supplemented with TPO cocktail (50 ng/ml TPO, 25 ng/ml SCF, 10 ng/ml IL-3, 10 ng/ml IL-6). To induce erythroid differentiation, CD34^+^ cells were cultured in SFEM medium that was supplemented with EPO cocktail (1 U/ml EPO, 25 ng/ml SCF, 30 μg/ml holo-transferrin, 10 nM β-mercaptoethanol). To induce megakaryocytic and erythroid differentiation simultaneously, CD34^+^ cells were cultured in SFEM that was supplemented with 25 ng/ml SCF plus all other components of the TPO and EPO cocktails indicated above. To induce megakaryocytic and erythroid differentiation of K562 cells, cells were cultured in RPMI 1640 medium that contained 10 nM PMA and 10 ng/ml ActA, respectively. Following culture for times indicated in the legends, cells were assayed for morphology and hemoglobin, and analyzed by flow cytometry as described below.

### Colony formation

To assay BFU-E, CFU-GM and CFU-GEMM formation, transduced CD34^+^ cells were sorted for GFP using a FACS AriaII cell sorter (BD Biosciences) and then seeded in low-adherence dishes that contained methylcellulose-based semi-solid medium supplemented with SCF, EPO, GM-CSF, G-GSF and IL-3 (MethoCult™ H4034 Optimum, Stem Cell Technologies). After 14 days, colonies were scored by standard morphologic criteria. Because CFU-Meg formation cannot be effectively resolved in methylcellulose-based medium, CFU-Meg formation was assayed by seeding GFP-sorted CD34^+^ cells in chamber slides that contained collagen-based semi-solid medium supplemented with SCF, TPO, IL-3, IL-6 and IL-11 (MegaCult™-C Complete Kit With Cytokines, Stem Cell Technologies). After 14 days, cells were fixed in methanol:acetone (1:3), stained with anti-CD41 antibody and colonies counted. Numbers of colonies originating from 10^4^ GFP-sorted cells were scored in each experiment and three independent experiments were performed.

### Cell morphology and adhesion assays

For evaluating cell morphology, cells were cytocentrifuged onto glass slides, fixed in methanol and stained with Wright-Giemsa solution (Fisher Scientific, Waltham, MA). For assaying cell adhesion, 4×10^5^ cells were plated in 35-mm fibronectin-coated dishes. At 16 h thereafter, floating cells in culture medium were counted using a hemocytometer. Adherent cells were removed by trypsin treatment, resuspended in fresh medium and counted.

### Flow cytometry

For cell surface staining, cells were incubated with antibodies to CD61, CD41, CD42a (1:10) or GYPA (1:20) for 30 min at 4°C, washed and incubated with peridinin-chlorophyll-protein complex (PerCP)-conjugated anti-mouse-IgG (1:5) for 30 min at 4°C. For intracellular staining, cells were fixed in 1% paraformaldehyde (20 min at 4°C) and permeabilized in 0.1% saponin (15 min at room temperature). Following washing, cells were incubated with antibodies to DLX4 (1:20) or phosphorylated p65 (1:500), washed and incubated with secondary antibodies. Thereafter, cells were washed and fixed in 4% paraformaldehyde. For dual staining, cells were incubated with fluorescein-conjugated CD42a antibody (1:11), then fixed, permeabilized and stained with DLX4 antibody as described above. To calculate absolute cell numbers, CountBright™ Absolute Counting Beads (Invitrogen) were added and evaluated according to manufacturer's instructions. To assay cell death, cells were stained with Annexin V (BD Biosciences) or 7AAD (Phoenix Flow Systems, San Diego, CA). To assay DNA content, cells were fixed in 70% ethanol, washed and then incubated with propidium iodide solution containing RNase A (Phoenix Flow Systems). Staining was detected by flow cytometry (FACS Calibur, BD Biosciences) and analyzed by CellQuest software (BD Biosciences). Three independent experiments were performed for each assay.

### Hemoglobin assay

Transduced CD34^+^ cells were sorted for GFP and then stimulated to undergo erythroid differentiation as described above. Hemoglobin was detected by staining with benzidine solution (2.92% v/v acetic acid, 0.2% w/v benzidine hydrochloride and 2.0% H_2_O_2_) at room temperature for 20 min and protected from light. Stained cells were viewed by light microscopy and counted in three random 100× fields per experiment. A minimum of 100 cells were evaluated in one field. Three independent experiments were performed.

### Luciferase assays

The NF-κB-LUC reporter construct (Cignal NF-κB reporter kit) was purchased from SABiosciences (Valencia, CA). Cells were co-transfected with firefly luciferase reporter plasmid, *DLX4* cDNA or shRNA plasmids and *Renilla* luciferase reporter plasmid, to normalize transfection efficiency, as previously described (Trinh et al., 2011). Luciferase activities were assayed using the dual-luciferase reporter assay kit (Promega, Madison, WI). Three independent experiments were performed for each assay.

### Quantitative reverse transcription PCR

Transcripts were analyzed by using SYBR^®^ Green qPCR Master Mix (SABiosciences). Primers to detect *DLX4*, *ITGA2B*, *GYPA*, *CXCL2*, *CXCL3*, *FOS*, *ICAM1*, *IL1A*, *IL8*, *JUN*, *LTA*, *TIMP1*, *TNF*, *TNFAIP3* and *TNFSF10* were from SABiosciences. *IL1B* primers were as follows: forward, 5′-CCACAGACCTTCCAGGAGAATG-3′; and reverse, 5′-GTGCAGTTCAGTGATCGTACAGG-3′. *RPL32* transcript levels were used as controls for normalization and were detected by using the following primers: forward, 5′-ACAAAGCACATGCTGCCCAGTG-3′; and reverse, 5′-TTCCACGATGGCTTTGCGGTTC-3′. Relative quantitation between samples and control transcript levels was performed by using the comparative Ct (2^−ΔΔCt^) method (Pfaffl, 2001).

### Chromatin immunoprecipitation

Chromatin immunoprecipitation assays were performed by using the EZ-ChIP Assay kit (Millipore, Billerica, MA). Sheared chromatin was incubated overnight with 1 µg DLX4 antibody. DNA was purified from precipitated complexes. A 316-bp fragment of the human *IL1B* promoter was amplified by using the following primers: forward, 5′-GGTAGAGACCCACACCCTCA-3′; and reverse, 5′-CATGGAAGGGCAAGGAGTAG-3′. As an irrelevant gene control, a 166-bp *GAPDH* fragment was amplified using the following primers: forward, 5′-TACTAGCGGTTTTACGGGCG-3′; and reverse, 5′-TCGAACAGGAGGAGCAGAGAGCGA-3′.

### Gene expression microarray analysis

Total RNA was extracted from cells using the PureLink™ RNA Mini kit (Ambion, Waltham, MA). Three independent RNA samples were prepared from each cell line. 500 ng of total RNA was used for labeling. Hybridization to HumanHT-12 v4 bead chips was performed following manufacturer's instructions (Illumina Inc., San Diego, CA). Bead chips were scanned with a BeadArray Reader (Illumina Inc). Microarray data were normalized by using the quantile normalization method in the Linear Models for Microarray Data (LIMMA) package within the R language environment (Wright and Simon, 2003). The expression level of each gene was transformed to a log_2_ scale prior to further analysis. Primary microarray data was deposited in the Gene Expression Omnibus (GEO) database (http://www.ncbi.nlm.nih.gov/geo) (microarray platform, GPL10558; microarray data, GSE63888). The gene expression data from Novershtern et al. (Novershtern et al., 2011) was downloaded from the GEO database (accession number GSE24759). Normalized data by the authors was used. The most variant probes were selected to represent the genes. Genes that were differentially expressed between two classes were identified by Biometric Research Branch (BRB) ArrayTools using a random-variance *t*-test (Simon et al., 2007). Differences in gene expression were considered to be statistically significant if the *P*-value was <0.001. A global test with 1000 permutations for evaluating the difference between gene expression profiles of two classes was performed by permuting the labels of which arrays corresponded to which classes. The proportion of the permutations that gave at least as many significant genes as with the actual data was the significance level of the global test. Cluster and heatmap analyses were performed by using Cluster and TreeView software (Eisen et al., 1998) where average expression values of samples within a given group were used.

### Gene ontology analysis

Gene Ontology analysis of biological functions was performed by using the Database for Annotation, Visualization and Integrated Discovery functional annotation tool (http://david.abcc.ncifcrf.gov). Significance of over-represented Gene Ontology biological processes was determined based on −log_10_ of corrected *P* values (Bonferroni-corrected modified Fisher's exact test) (Dennis et al., 2003).

### Gene Set Enrichment Analysis

GSEA was performed using GSEA v2.1.0 software (Subramanian et al., 2005). Gene sets were scored using the signal-to-noise ratio in the weighted enrichment score. *P* values were calculated by using 1000 permutations of the phenotype. Curated gene sets from the Molecular Signatures Database of the Broad Institute (http://www.broadinstitute.org/gsea/msigdb/index.jsp) were used. Megakaryocyte gene sets were from the studies of Mercher et al. (Mercher et al., 2008) and Tenedini et al. (Tenedini et al., 2004). Erythroid gene sets were from the studies of Ebert et al. (Ebert et al., 2008) and Steiner et al. (Steiner et al., 2009). The megakaryocyte gene set that we generated from the study by Novershtern et al. was defined as the top 333 genes having significantly higher level of expression in mature megakaryocytes (MEGA2) as compared to short-term HSCs (HSC2) (Novershtern et al., 2011). The erythroid gene set that we generated from the study by Novershtern et al. was defined as the top 260 genes having significantly higher level of expression in late erythroid cells (ERY5) as compared to short-term HSCs (HSC2) (Novershtern et al., 2011).

### Statistical analysis

Statistical significance of data was assessed by unpaired two-tailed Student's *t-*test by using STATISTICA10 software (StatSoft Inc., Tulsa, OK). *P* values of <0.05 were considered significant.

## Supplementary Material

Supplementary Material
