# Supplementary material for: The homeobox gene DLX4 regulates erythro-megakaryocytic differentiation by stimulating IL-1β and NF-κB signaling
Source: J Cell Sci. 2015 Aug 15;128(16):3055–67. doi: 10.1242/jcs.168187 (PMC4541043; doi:10.1242/jcs.168187)
Supplement: Supplementary Material [file supp_128_16_3055__index.html]

Supplementary Material 

# The homeobox gene *DLX4* regulates erythro-megakaryocytic differentiation by stimulating IL-1/NF-κB signaling

## JCS168187 Supplementary Material

- Supplementary Material
