# Supplementary material for: The homeobox gene DLX4 regulates erythro-megakaryocytic differentiation by stimulating IL-1β and NF-κB signaling
Source: J Cell Sci. 2015 Aug 15;128(16):3055–67. doi: 10.1242/jcs.168187 (PMC4541043; doi:10.1242/jcs.168187)
Supplement: Supplementary Material [file supp_jcs.168187_JCS168187supp.pdf]

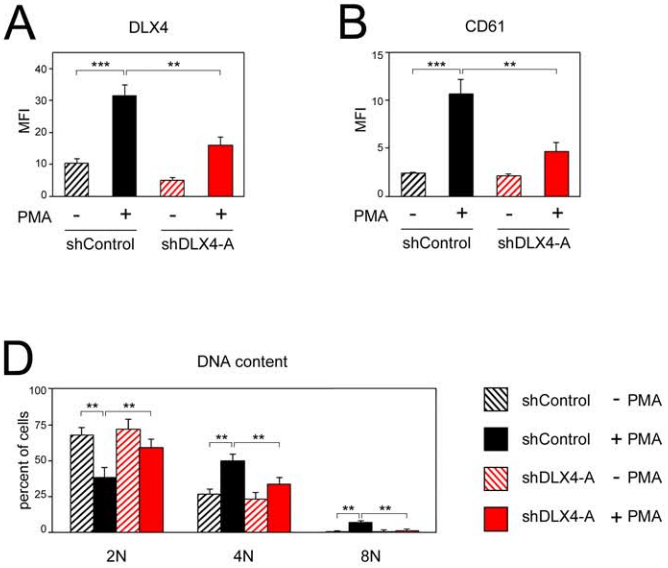

Fig. S1. Knockdown of DLX4 prevents megakaryocytic differentiation of K562 cells. K562 cells that were transfected with non-targeting shRNA (shControl) or with *DLX4* shRNA (shDLX4-A) were treated with DMSO (-PMA) or with 10 nM PMA (+PMA). At 3 days thereafter, cells were evaluated by flow cytometry for **(A)** intracellular staining of DLX4 and for cell surface staining of **(B)** CD61 and **(C)** CD41. Shown are average MFI of staining of three independent experiments. **(D)** Cells were stained with propidium iodide and analyzed for DNA content by flow cytometry. Shown are average percentages of cells with different ploidy of three independent experiments. $^{**}P < 0.01$, $^{***}P < 0.001$.

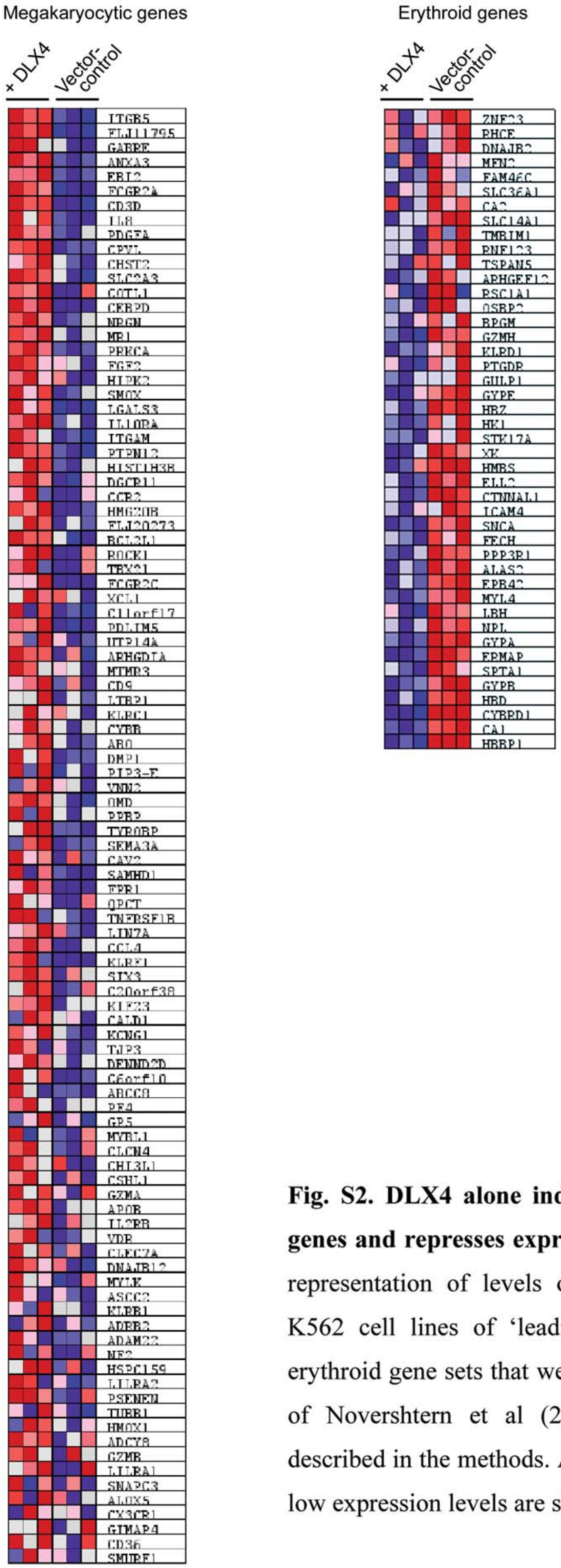

Fig. S2. DLX4 alone induces expression of megakaryocyte-specific genes and represses expression of erythroid-specific genes. Heat map representation of levels of expression in vector-control and +DLX4 K562 cell lines of 'leading edge' genes of the megakaryocyte and erythroid gene sets that were generated from the gene expression dataset of Novershtern et al (2011) (GEO Accession No. GSE24759) as described in the methods. Analysis was performed in triplicate. High and low expression levels are shown in red and blue, respectively.

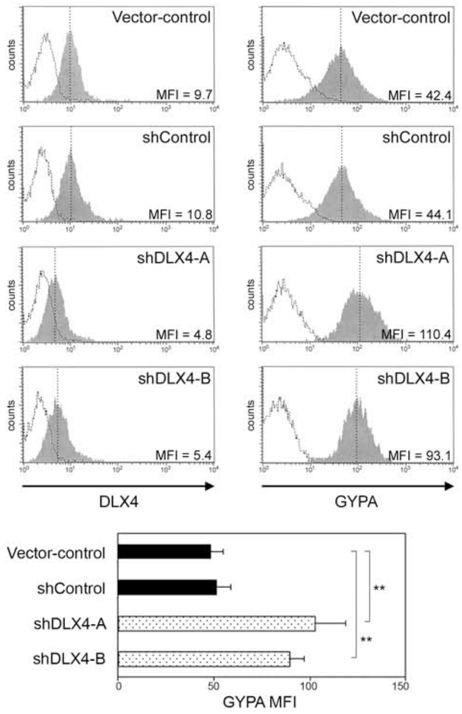

Fig. S3. DLX4 inhibits erythroid differentiation.

Staining of DLX4 and GYPA were evaluated in vector-control K562 cells and K562 cells transfected with non-targeting shRNA (shControl) and *DLX4* shRNAs (shDLX4-A, shDLX4-B). *Upper panels*, representative examples of flow cytometric analysis of staining. Solid grey histograms represent staining with Abs to DLX4 and to GYPA with MFI indicated. Dotted histograms represent staining with isotype control. *Lower panel*, average MFI of GYPA staining of three independent experiments. $^{**}P < 0.01$.

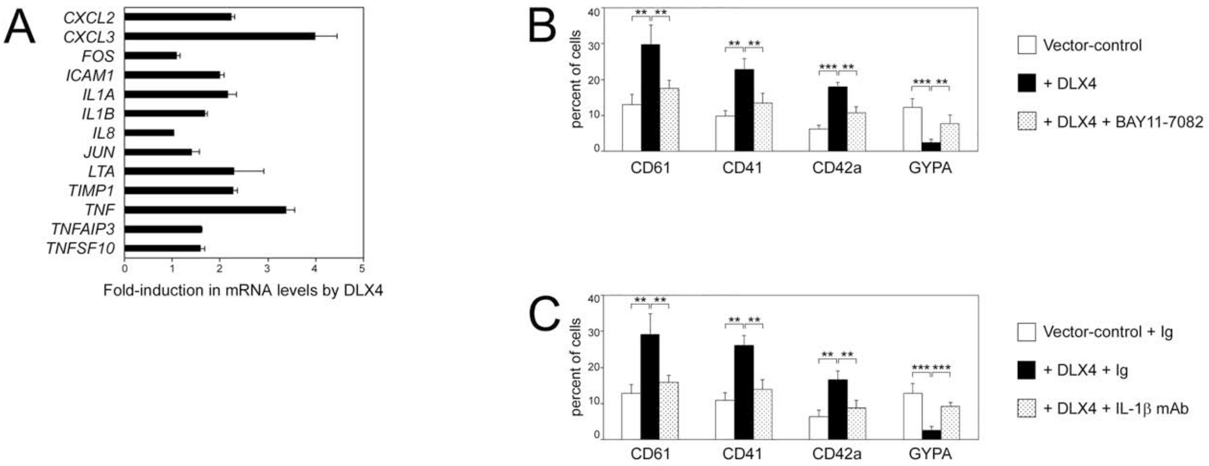

Fig. S4. DLX4 controls megakaryocytic and erythroid differentiation of $CD34^+$ cord blood cells in a NF-κB- and IL-1β- dependent manner. (A) $CD34^+$ cord blood cells were transduced with GFP-expressing vector-control and DLX4 lentiviruses, sorted for GFP and then analyzed for expression of NF-κB target genes by qRT-PCR. Shown is the average fold-induction in the mRNA level of each gene in +DLX4 cells relative to its respective level in vector-control cells. In B and C, transduced $CD34^+$ cells were incubated with medium that contained both EPO and TPO cocktails, and with or without the addition of **(B)** the IκB kinase inhibitor BAY11-7082 (1 μM) and **(C)** control Ig or neutralizing mAb to IL-1β (1 μg/mL). At 5 days thereafter, staining of CD61, CD41, CD42a and GYPA was evaluated within the gated population of transduced GFP^+ cells by flow cytometry. Shown in A, B and C are mean $\pm$ s.d. values of three independent experiments. $^{**}P < 0.01$, $^{***}P < 0.001$.

Table S1: DLX4 transcriptional program is enriched for gene sets associated with cell adhesion

GSEA gene sets	No. of genes	NES	*P* value	FDR q value
CELL SUBSTRATE ADHESION	39	1.57	< 0.001	0.133
CELL MATRIX ADHESION	38	1.55	< 0.001	0.133
EXTRACELLULAR MATRIX	100	1.53	< 0.001	0.106
BOWIE RESPONSE TO EXTRACELLULAR MATRIX	17	1.52	<0.001	0.093
PROTEINACEOUS EXTRACELLULAR MATRIX	98	1.52	< 0.001	0.085
EXTRACELLULAR MATRIX PART	57	1.49	< 0.001	0.080
REACTOME PLATELET ADHESION TO EXPOSED COLLAGEN	11	1.44	< 0.001	0.103
REGULATION OF CELL CELL ADHESION	10	1.36	<0.001	0.115
REACTOME EXTRACELLULAR MATRIX ORGANIZATION	87	1.30	<0.001	0.188

Global changes in gene expression that are induced by DLX4 in K562 cells were evaluated by GSEA for enrichment for cell adhesion gene sets in the Molecular Signatures Database of the Broad Institute. Normalized enrichment scores (NES), significance values and false discovery rates (FDR) are indicated.

Table S2: DLX4 transcriptional program is enriched for gene sets associated with NF-κB signaling

GSEA gene set	No. of genes	NES	*P* value	FDR q value
DUTTA APOPTOSIS VIA NFKB	32	1.58	< 0.001	0.106
REACTOME P75NTR SIGNALS VIA NFKB	13	1.56	< 0.001	0.077
PID NFKAPPAB ATYPICAL PATHWAY	17	1.55	< 0.001	0.067
REACTOME NFKB IS ACTIVATED AND SIGNALS SURVIVAL	10	1.53	< 0.001	0.068
BIOCARTA NFKB PATHWAY	22	1.47	< 0.001	0.191
BIOCARTA EPONFKB PATHWAY	11	1.46	< 0.001	0.179
HINATA NFKB TARGETS FIBROBLAST UP	83	1.42	< 0.001	0.228
REACTOME TRAF6 MEDIATED INDUCTION OF NFKB AND MAP KINASES UPON TLR7 8 OR 9 ACTIVATION	72	1.42	< 0.001	0.224
REACTOME TAK1 ACTIVATES NFKB BY PHOSPHORYLATION AND ACTIVATION OF IKKS COMPLEX	20	1.35	< 0.001	0.220
REACTOME NFKB ACTIVATION THROUGH FADD RIP1 PATHWAY MEDIATED BY CASPASE 8 AND 10	11	1.34	< 0.001	0.191
REACTOME NFKB AND MAP KINASES ACTIVATION MEDIATED BY TLR4 SIGNALING REPERTOIRE	67	1.29	< 0.001	0.214
HINATA NFKB TARGETS KERATINOCYTE UP	91	1.29	< 0.001	0.206
REACTOME RIP MEDIATED NFKB ACTIVATION VIA DAI	18	1.24	< 0.001	0.206
HINATA NFKB MATRIX	10	1.17	< 0.001	0.224

Global changes in gene expression that are induced by DLX4 in K562 cells were evaluated by GSEA for enrichment for gene sets that are associated with NF-κB signaling in the Molecular Signatures Database of the Broad Institute. Normalized enrichment scores (NES), significance values and false discovery rates (FDR) are indicated.
